# Function of a Fly Motion-Sensitive Neuron Matches Eye Movements during Free Flight

**DOI:** 10.1371/journal.pbio.0030171

**Published:** 2005-05-17

**Authors:** Roland Kern, J. H van Hateren, Christian Michaelis, Jens Peter Lindemann, Martin Egelhaaf

**Affiliations:** **1**Department of Neurobiology, Faculty for BiologyBielefeld University, BielefeldGermany; **2**Department of NeurobiophysicsUniversity of GroningenThe Netherlands; Australian National UniversityAustralia

## Abstract

Sensing is often implicitly assumed to be the passive acquisition of information. However, part of the sensory information is generated actively when animals move. For instance, humans shift their gaze actively in a sequence of saccades towards interesting locations in a scene. Likewise, many insects shift their gaze by saccadic turns of body and head, keeping their gaze fixed between saccades. Here we employ a novel panoramic virtual reality stimulator and show that motion computation in a blowfly visual interneuron is tuned to make efficient use of the characteristic dynamics of retinal image flow. The neuron is able to extract information about the spatial layout of the environment by utilizing intervals of stable vision resulting from the saccadic viewing strategy. The extraction is possible because the retinal image flow evoked by translation, containing information about object distances, is confined to low frequencies. This flow component can be derived from the total optic flow between saccades because the residual intersaccadic head rotations are small and encoded at higher frequencies. Information about the spatial layout of the environment can thus be extracted by the neuron in a computationally parsimonious way. These results on neuronal function based on naturalistic, behaviourally generated optic flow are in stark contrast to conclusions based on conventional visual stimuli that the neuron primarily represents a detector for yaw rotations of the animal.

## Abstract 2

With the aid of virtual reality, the authors study the neuronal responses to visual motion associated with natural behavior.

## Introduction

In moving animals, retinal image flow differs from conventional visual stimuli used in the laboratory, by its characteristic dynamics that are largely determined by the animals' own actions and reactions. For instance, the retinal image flow on the eyes of humans steering a car, is determined by the direction and speed of the car, but also by the body, head, and eye movements of the driver. In particular, the eyes are rotated actively in a sequence of saccades towards interesting locations in the scene (review [1]). Many insects, such as blowflies, employ a similar saccadic viewing strategy (review [2]). They shift their gaze during free flight by saccadic turns of body and head, keeping gaze basically fixed between saccades [3–5]. This active viewing strategy generates retinal image flow with characteristic dynamical features and separates to a large extent the image flow resulting from rotational and translational movements of the animal. Because the rotational optic flow component does not depend on the distance between the eyes and environmental objects, whereas the translational flow component does, the saccadic flight strategy may help the nervous system to extract information about the spatial layout of the environment. So far, it is not clear to what extent neuronal processing matches the specific properties of the retinal image flow during natural behaviour and thus may be appropriate for gathering environmental information. It is possible that the mechanisms of neuronal information processing are specifically adapted to efficiently utilize image flow under behaviourally relevant conditions. This hypothesis is tested here by analysing the performance of an identified motion-sensitive neuron in the blowfly under stimulus conditions that approximate natural situations.

To circumvent the problems of recording neuronal responses in freely moving animals we took advantage of recent technological developments: In the blowfly, a model system for visual motion computation [6,7], body and even head movements were recorded during free flight [5,8]. The resulting behaviourally generated retinal image flow was reconstructed (Video S1) and replayed to blowflies with a panoramic visual stimulator that is sufficiently fast to show visual stimuli as experienced even during rapid saccadic turns [9]. During replay the activity of the so-called horizontal system equatorial cell (HSE) [10,11] was recorded intracellularly.

**Figure 1:**
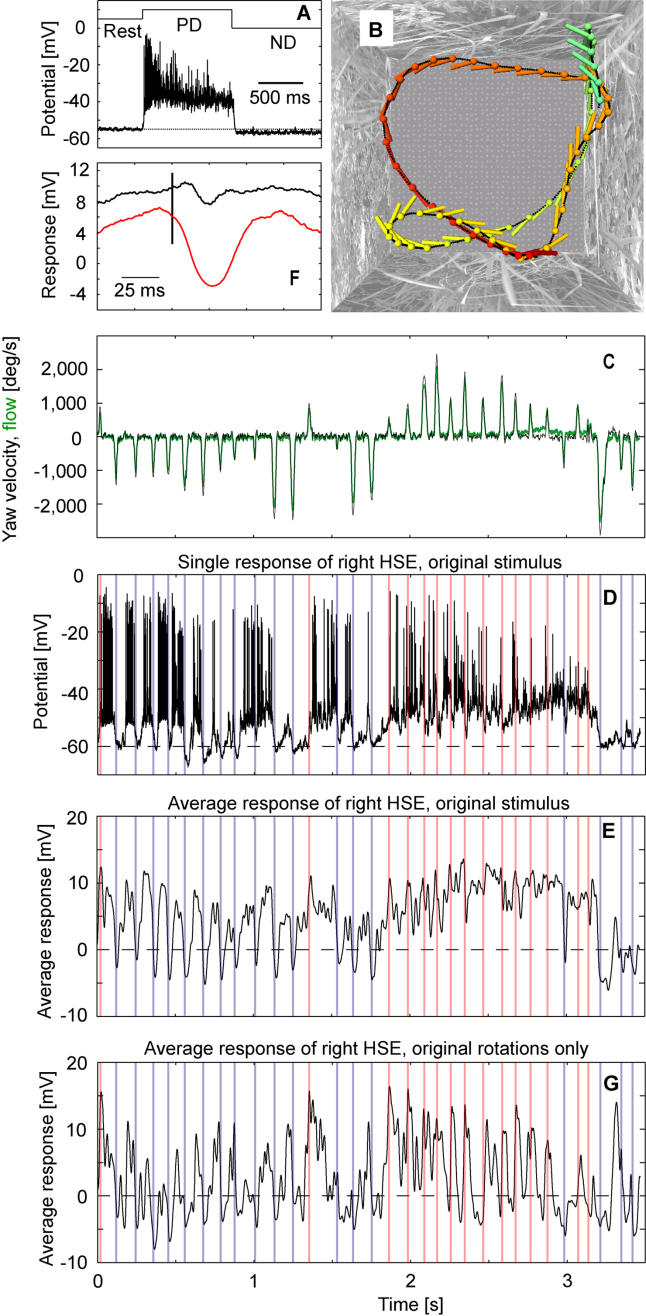
Response Characteristics of HSE under Various Stimulus Conditions (A) Individual response of the right HSE to rightward (preferred-direction [PD]) and leftward (null-direction [ND]) rotation of a vertical sinusoidal grating (wavelength 20 deg, velocity 40 deg/s, contrast 0.98, azimuth ±120 deg, elevation ±50 deg). (B) Downward view of a flight trajectory (dotted line), with head position and orientation shown every 50 ms (time colour coded: start, red; finish, green). (C) Head yaw velocity (black) and total optic flow (green) during the flight shown in (B). Positive velocities denote leftward turns, PD for the right HSE. (D) Membrane potential of a right HSE in response to the flight shown in (B). (E) Average membrane potential of the right HSE as determined across trials; the entire responses including spikes were taken into account (*N* = 6 cells, *n* = 17 responses). (F) Saccade-triggered averages, calculated from the mean responses (*N* = 6, one to four repetitions) of right and left HSE to two trajectories for PD (black) and ND (red) saccades (63 different saccades). Vertical line denotes time of saccade peak velocity. (G) Same as (E), but with rotations only (position of fly fixed in centre of cage; *N* = 6, *n* = 11). Broken lines denote resting potential; red or blue vertical lines indicate peak times of PD or ND saccades, respectively. Signals in (D), (E), and (G) are shifted backwards by 22.5 ms to account for response latencies; this value was determined by cross-correlation of the yaw velocity and the corresponding response traces. Signals in (E) and (G) are low-pass filtered (with a Gaussian standard deviation of 3 ms).

HSE is a major output neuron of the visual system and belongs to an identified set of motion-sensitive neurons present in both the left and right third visual neuropil of the blowfly brain. These neurons are believed to extract parameters of self-motion from the optic flow field [10–12]. HSE responds in a directionally selective manner to visual wide-field motion; because of its input from many local motion-sensitive elements, it is depolarised by front-to-back motion in the ipsilateral visual field and hyperpolarised during motion in the reverse direction [6]. The graded depolarisations, although superimposed by spikes of variable amplitude, are still quite pronounced in the axon terminal of the cell [10,11,13] (Figure 1A). Such depolarisations were shown in other motion-sensitive neurons with the same type of mixed response to be transmitted to postsynaptic cells [14]. Because HSE also receives excitatory input from the contralateral eye during back-to-front motion via the identified H1 and H2 neurons, previous studies using conventional experimenter-defined stimuli concluded that its main functional role is to encode rotations around the vertical axis of the head (yaw). In addition, HSE also responds to binocular front-to-back motion [10,12,15], as occurs during forward flight.

Responses to behaviourally generated optic flow, however, cast doubt on the presumed role of HSE. We provide evidence that HSE, rather than primarily representing a detector for yaw rotations of the animal, also encodes information about sideward translational optic flow. This feature could be significant from a functional point of view because only optic flow induced by translatory motion, but not by rotatory motion, contains information about the spatial layout of the environment. The animal's saccadic flight style is concluded to produce a match of the dynamical properties of retinal image flow with the visual motion pathway of the blowfly.

## Results

The velocity profile of yaw rotations reflects the blowfly's saccadic flight style: The fly executes a series of saccadic turns in which the head shows peaks in yaw velocity approaching several thousand degrees per second (Figure 1B and 1C). Between saccades the gaze is kept basically stable and the resulting optic flow is much smaller than during saccades (Figure 1C). Figure 1D shows a recording from an HSE cell of a blowfly watching a movie, played back on the panoramic stimulator, consisting of the image flow experienced by another blowfly during the flight of Figure 1B. For most of the time during this stimulus, the HSE cell was depolarised relative to its resting potential. This depolarisation was interrupted by brief hyperpolarisations during saccades evoking null-direction motion (“null-direction saccades”) (Figure 1D and 1E, blue lines; Figure 1F, red trace). In contrast to expectations from responses to conventional stimuli, HSE did not consistently depolarise during saccades leading to preferred-direction motion (“preferred-direction saccades”), but often showed a slight response dip (negative-going response) relative to the ongoing depolarisation level (Figure 1D and 1E, red lines; Figure 1F, black trace). Similar response profiles were obtained for all ten behaviourally generated motion sequences that were tested. Hence, we may conclude that the overall depolarisation of HSE is not evoked by preferred-direction saccades, but by optic flow *between* saccades.

**Figure 2:**
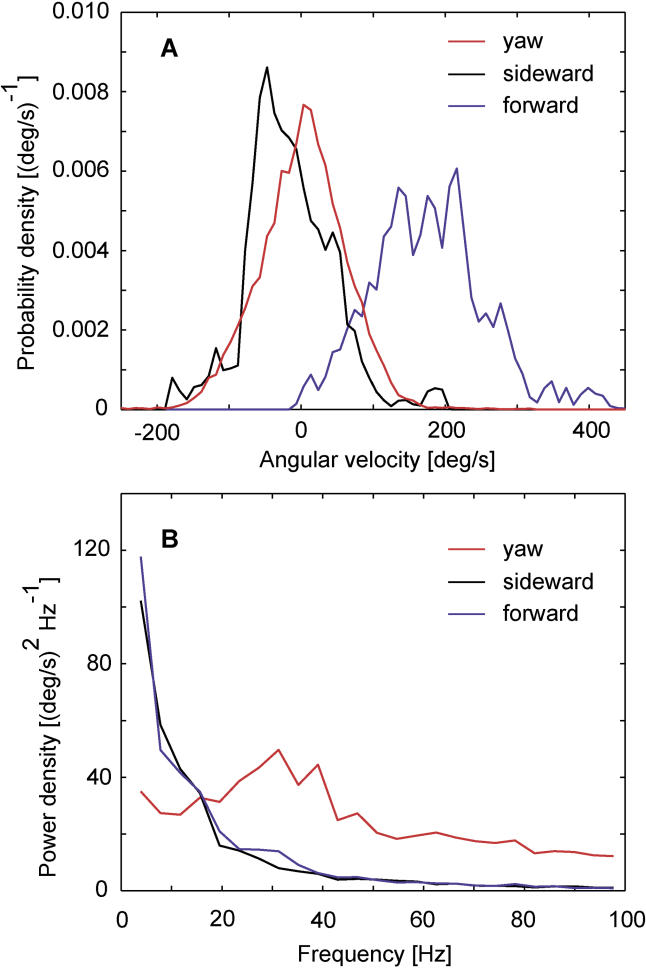
Properties of the Intersaccadic Stimulus for the Trajectories Used for Figure 3A (A) Probability density function of yaw velocity (red), sideward (black), and forward (blue) velocity. Forward is parallel to the frontal axis of the fly's head; sideward is perpendicular to the head's plane of symmetry. Sideward and forward velocities were converted to angular velocities by multiplying by the nearness (equal to the inverse of the distance [16]) averaged over the trajectories and over the receptive fields of the neurons (7.02 m^−1^). (B) Power spectra of yaw velocity (red), sideward (black), and forward (blue) angular velocity. Typical relative standard error of the mean (SEM) = 20%.

This finding was surprising because the strongest optic flow in the cell's preferred direction was generated during preferred-direction saccades rather than between saccades. The possibility that the angular velocities during saccades were too large to be perceived by the motion vision system can be excluded. When the neuron was stimulated exclusively with the original rotations without any superimposed translation, thereby mimicking a situation in which the environmental structures were at an infinite distance (Figure 1G), pronounced depolarising response peaks during preferred-direction saccades occurred. From the considerable difference between the responses of Figure 1E and 1G, it follows that the translational optic flow component has a major impact on the neuronal response profile, even though the translational component is much smaller than the rotational component evoked by saccades. Because blowflies keep their gaze stable between saccades, apart from small-amplitude, broad-band yaw rotations (Figures 1C, 2A, and 2B), they may gather useful information about the spatial layout of the outside world from the translational optic flow components that dominate at low frequencies in intersaccadic intervals.

**Figure 3:**
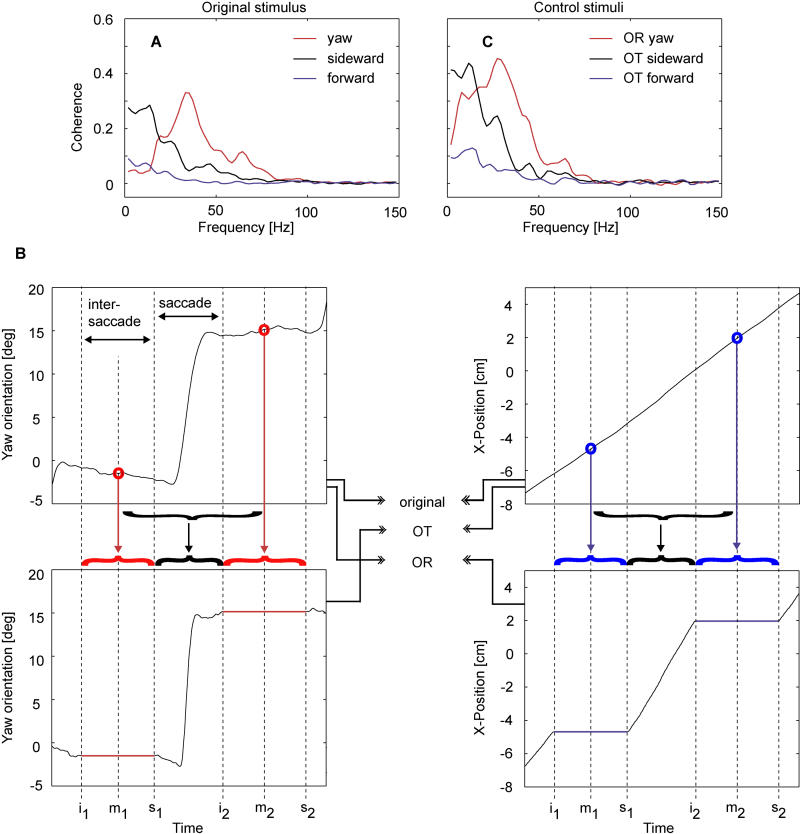
Coherence of Stimulus Parameters with HSE Response, Intersaccadic Parts of Stimulus and Response Only (A) Average coherence of the response of the right and left HSE (*N* = 5 cells) with yaw velocity (red), sideward (black), and forward (blue) velocity. (B) Construction of control stimuli. Times *i* denote the start of an intersaccadic period, *m* is its midpoint, and *s* is the start of a saccade. For OT, each orientation coordinate (yaw shown) between consecutive midpoints is compressed into the saccadic period, leaving orientation constant between saccades. For OR, each position coordinate (*x* shown) is similarly compressed, leaving position constant between saccades. (C) Average coherence of yaw velocity for the OR (right and left HSE, *N* = 2) control (red), and of sideward (black) and forward (blue) velocity for the OT (*N* = 3) control. Typical relative SEM = 10% for all coherences shown at (A) and (C).

To test this hypothesis, we analysed the intersaccadic-response segments by masking the saccadic segments of stimulus and response. Masks were obtained by gating a region surrounding each saccade that was large enough to include all parts of both saccadic stimulus and corresponding response (for details see Materials and Methods). To establish whether single HSE responses provide the animal with information on its self-motion parameters that could be accessed by simple filtering operations, we determined the optimal linear filters by estimating these parameters from the responses. The similarity between estimated and original self-motion parameters was quantified by the coherence that varies between zero (i.e., both signals are unrelated) and one (i.e., perfect reconstruction). Whereas the coherence of the intersaccadic yaw velocity and the neuronal response was significant only between approximately 20 Hz and 60 Hz, there was considerable coherence between sideward velocity and the neuronal responses at low frequencies (Figure 3A, results from ten flights and five HSE neurons). Surprisingly, the coherence was much smaller for the forward velocity although HSE responds well to constant-velocity front-to-back motion [10,11,15] (see Figure 1A). The coherence with the other self-motion parameters (upward velocity, pitch, and roll) was negligible. We conclude from these results that HSE might make use of the difference in frequency content of rotations and translations (see Figure 2B) to provide information on both optic flow components in adjacent frequency bands (Figure 3A). This is possible because the intersaccadic yaw velocities are smaller by an order of magnitude than during saccades (compare Figures 1C and 2A). Hence, the saccadic gaze strategy may be viewed as a specialisation that enables the extraction of translatory optic flow amidst rotatory optic flow that would otherwise dominate the response if smooth yaw rotations were used exclusively for steering.

The intersaccadic responses to rotation and translation during natural flight might interfere with each other in a complicated way. To check whether such an effect influences our conclusions, we designed two control stimuli (Figure 3B and Video S2). These control stimuli allowed us to study the response to rotation and translation separately whilst keeping the visual scene viewed by the fly virtually the same. In the only rotation (OR) control there was no translation between saccades, so to obtain basically the same trajectory of the eye, the intersaccadic translation of the original trajectories was added to the translation during saccades. In contrast, in the only translation (OT) control, the eye was assumed to translate without any superimposed rotation between saccades; the rotation between saccades was added to saccadic rotation. Coherence of yaw velocity with the response to OR stimuli and coherence of the translational velocities with the response to OT stimuli show similar frequency dependencies as the corresponding components of the original optic flow (Figure 3A and 3C), confirming our conclusion of a frequency separation of the rotational and translational components. Again, the coherence was much smaller for the forward velocity than for the sideward velocity. Hence, the low-frequency components of the neuronal responses between saccades encode mostly sideward motion, whereas information about yaw velocity dominates the high-frequency response components.

**Figure 4:**
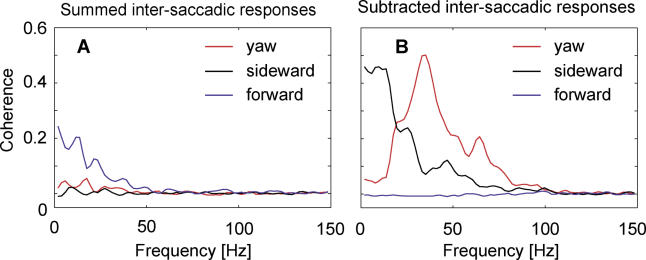
Coherence of Stimulus Parameters with Combined HSE Responses, Intersaccadic Parts of Stimulus and Response Only (A) Average coherence of yaw (red), sideward (black), and forward (blue) velocity with the summed responses of right and left HSE (*N* = 5). (B) Same as (A) for the subtracted responses of right and left HSE. Typical relative SEM = 10% for all coherences shown at (A) and (B).

By combining the responses of the HSE cells from both halves of the brain, the specificity of the intersaccadic responses to the translational optic flow components can be enhanced. The summation of the responses almost exclusively signifies forward velocity (Figure 4A), whereas the difference between the responses almost exclusively signifies the sideward and yaw velocities (Figure 4B). The latter signals can be separated by low-pass and band-pass filtering, respectively. It is not known whether the blowfly actually uses such a processing scheme, but it is clear that the information on yaw, forward, and sideward velocity can be extracted by simple operations that can also be interpreted in neuronal terms.

**Figure 5:**
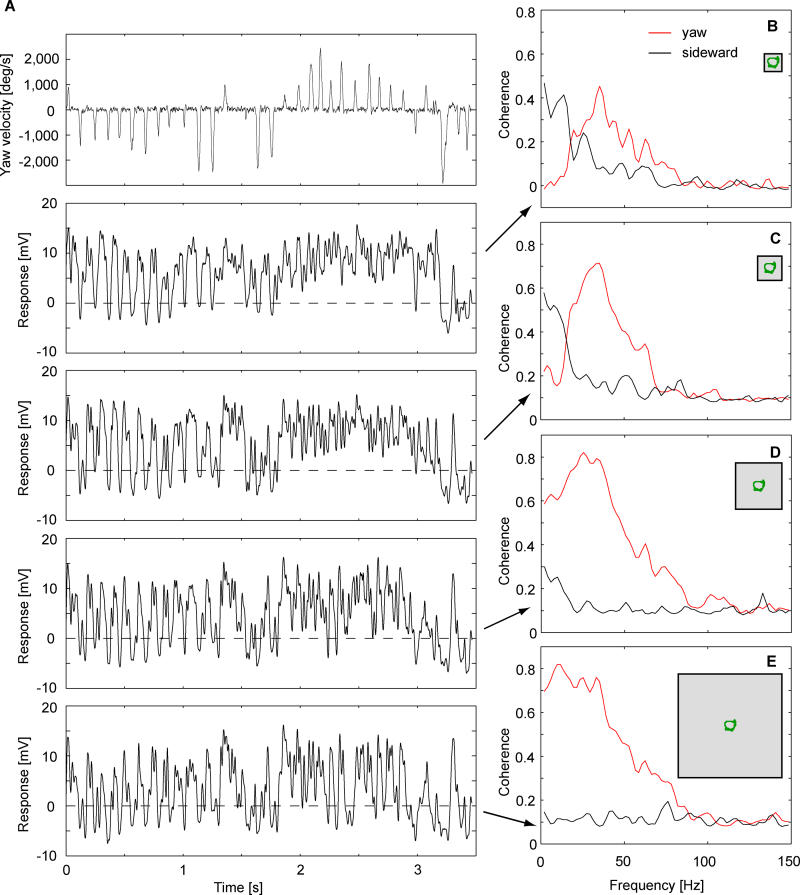
Influence of Wall Nearness (A) Yaw velocity and average responses (*n* = 4, low-pass filtered with a Gaussian standard deviation of 3 ms) to behaviourally generated optic flow of a given flight trajectory in the original flight arena but also in virtual flight arenas with increasingly larger size. (B–E) Coherence of yaw velocity (red) and sideward angular velocity (black) for different cage sizes: 40-cm edge length (original cage; B), 55 cm (C), 105 cm (D), and 235 cm (E). For (C–E) the flight was centred in the cage. Insets in (B–E): the original and the virtual arenas as seen from above.

Because translational optic flow depends on the distance of the animal to objects in its environment [16–18], the dependence of the neuronal responses on translation velocity is likely to reflect the spatial relation of the animal to its surroundings. This prediction is supported by experiments in which the optic flow of a given flight trajectory was tested not only for the original flight arena. but also for virtual flight arenas of increasingly larger size (Figure 5). When enlarging the virtual arena, the overall response profile changed dramatically and became virtually indistinguishable from the response to the original rotations without any superimposed translation (compare bottom trace in Figure 5A and Figure 1F) when the size of the flight arena increased to more than approximately 2 m. Accordingly, the coherence between the difference of the responses of the right and the left HSE and sideward velocity dropped to chance levels with increasing distance of the fly to the arena walls (Figure 5B–5E). Hence, intersaccadic responses of HSE implicitly reflect distance information. For the translatory velocities of flies observed in the present experiments in the original 40-cm cage, all distances larger than about 1 m were effectively at infinity.

## Discussion

Here it is shown that, between saccades, the neuronal signals of an identified motion-sensitive visual interneuron of the blowfly provide information about translatory self-motion and thus, implicitly, about the spatial relation of the animal to its surroundings. This result was obtained by a novel experimental paradigm that made it possible, for the first time, to present in electrophysiological experiments what an animal has seen during free-flight manoeuvres. Although the behavioural free-flight sequences were obtained in a relatively small flight arena, there are preliminary results that blowflies under natural outdoor conditions [19] employ the same saccadic flight strategy as observed under laboratory conditions. However, whereas the position and orientation of the eyes of free-flying blowflies could be resolved with unprecedented resolution in the laboratory setting by using a magnetic coil technique [4,5], reconstruction of gaze with a similar precision is not easily possible from high-speed video data collected outdoors. Hence, the behaviourally generated optic flow sequences that were used for stimulation in the present study represent the currently most precise approximation of the visual input of freely flying blowflies.

Our conclusions obtained with behaviourally generated optic flow do not match previous conclusions based on conventional stimuli exclusively defined by the experimenter.

In contrast to the common view that the analysed HSE neuron mainly acts as a detector for the animal's self-rotation [12,20], our results show that, depending on the three-dimensional layout of the environment, its response may not be dominated by the most prominent turns of the animal that occur during saccades.

Although the cell experiences the largest optic flow during saccades, it may encode behaviourally relevant information especially between saccades. Because blowflies keep their gaze stable between saccades apart from small, broad-band yaw rotations, they may gather useful information about the outside world from the translational optic flow components that dominate at low frequencies in intersaccadic intervals. Indeed, between saccades, neuronal signals provide rich information about the spatial relation of the animal to its surroundings. It should be noted that distance is signalled only relative to the fly's own velocity, because retinal velocities evoked during translation are inversely proportional to distance and proportional to translation velocity. This implies that in walking flies, the visual surroundings should affect the responses of the HSE cell only when the fly is very close to environmental structures, just as has been found previously [21,22]. This implicit scaling of distance information by the actual speed of the animal may be a parsimonious and advantageous way to extract from optic flow behaviourally relevant information about the outlay of the environment, because, for instance, evasive actions evoked by obstacles in the path of locomotion need to be evoked only at a smaller distance when the animal moves slower.

Based on experimenter-designed motion stimuli, motion-sensitive neurons are conventionally expected to encode stimulus velocity. Indeed, stimulus velocity can be reconstructed faithfully from the responses of blowfly motion-sensitive neurons as long as the velocities and velocity changes are relatively small [23,24]. However, during saccades the visual motion system operates far beyond the linear range. At higher velocities and, in particular, for very transient motion stimuli, the responses of motion-sensitive neurons are no longer determined by pattern velocity alone, but acceleration and higher time-derivatives of velocity presumably also play a role in shaping the response profile [25–28]. Hence, to assess the functional significance of neuronal mechanisms it is important to analyse the system under its natural operating conditions. The limited linear operating range of motion vision is frequently regarded as a disadvantage because motion-sensitive neurons are implicitly expected to encode velocity in a linear way. In contrast to this view, our results suggest that the nonlinearities of the visual motion system may be essential for HSE to encode information about the spatial relation of the animal to its environment. This interpretation is corroborated by model simulations of the blowfly's visual motion pathway and of HSE responses to behaviourally generated optic flow (J. P. Lindemann et al., unpublished data). If the neuron encoded linearly the entire velocity range that the system encounters in behaviour, by far the largest responses would be generated during body saccades. This would leave only a small response range for encoding information about optic flow between saccades. This information would be strongly degraded by noise in the neuronal signals. Hence, because during saccades the motion vision system does operate outside its linear range, it appears to be able, between saccades, to encode useful information about translation and thus about the spatial relation of the animal to the outside world.

How can the time-dependent responses to complex dynamic stimuli a blowfly encounters in free flight be explained? All features of the HSE responses that are characterised here by electrophysiological techniques can be explained by a model of the computational mechanisms implemented by the neuronal circuits in the blowfly motion vision pathway (J. P. Lindemann et al., unpublished data). Although this model was originally proposed based on simple experimenter-designed stimuli, we showed that it also exploits the active saccadic gaze and flight strategy of blowflies in a similar way to its neuronal counterpart. By stepwise dissection of the model circuit, we could determine which of its components are essential for these remarkable features. Most relevant is the nonlinear velocity encoding of the mechanism of local motion detection, modelled by correlation-type movement detectors, as well as the nonlinear spatial integration properties of the HSE cell itself. The model study suggests that the complex time course of the responses to behaviourally generated optic flow is not significantly shaped by adaptive processes. Such processes were previously characterised in various neurons, not only with simple constant velocity stimuli [29–35], but also with experimenter-designed time-varying velocity fluctuations [28,36,37]. The only adaptive change required in the model was a slight decrease of the gain of the system during prolonged stimulation with behaviourally generated optic flow. This finding of our modelling study is in accordance with recent experimental evidence [33,38].

The close similarity between the present electrophysiological results and the results of our model simulations (J. P. Lindemann et al., unpublished data) indicates that down to the level of the lobula plate the responses are primarily stimulus-driven (“bottom up”). It appears then that feedback mediated by other sensory modalities, such as from the haltere system [39–42] or efference copies, related, for example, to intended neck muscle activity (“top down”), are not important at this level of optic flow processing. Notwithstanding, it is conceivable that haltere signals or efference copies are advantageous when it comes to reading out the lobula plate signals, in particular when splitting these up into saccadic and intersaccadic intervals.

Although our experiments demonstrate that the fly's nervous system might be capable of extracting both rotational and translational information from the combined output of the HSE cells, this should not be taken as evidence that it does so. It is not known, so far, what features are extracted by downstream circuitry from the signals of HSE cells and of other motion-sensitive output neurons of the visual system. Subtraction or addition of the outputs of HSE cells from both halves of the visual system as done in our analysis are merely simple processing schemes that make information on yaw, forward, and sideward velocity available. Nonetheless, these simple formal operations can be approximated by cellular computations through combining excitatory and/or inhibitory synapses. Moreover, because synapses are often found to act as frequency filters (e.g., [43–45]), it appears to be feasible, by neuronal mechanisms, to separate the information on sideward translation and yaw rotation that is inherent in different frequency bands in the HSE difference signal. It will be of interest in future studies to determine whether the proposed encoding scheme, which the current experiments show is possible, is actually employed by the fly's brain during flight. Moreover, it needs to be worked out how the saccadic flight and gaze control system interacts with the system that mediates compensatory optomotor responses by reducing the slip velocity between the animal and its environment that results from unintended course deviations [46–48]. Such deviations may result, for instance, from asymmetries of the fly's motor system. The different dynamical properties of both systems would explain why saccadic turns are not counteracted by the compensatory optomotor system: Actively induced fast saccadic turns are not impeded by the much slower optomotor system [48,49]. There is evidence for this interpretation from recent behavioural experiments both on walking [50] and flying blowflies (R. K., unpublished data).

Our results suggest that the computational design principles of the blowfly visual motion pathway are adapted to the active vision strategies of the animal and therefore allow the extraction of behaviourally relevant information. Information resulting mainly from sideward movements of the animal in the intersaccadic interval may be used to elicit saccades that prevent the animal from crashing into an obstacle. Indeed, there is evidence from combined behavioural and modelling analyses on *Drosophila* that image expansion in the lateral visual field may play this important role [51].

In conclusion, our results provide an example in which a novel functional role of a neuron emerges by probing the neuron with stimuli that are actively generated by the animals' own behaviour. The new role is revealed here because the behaviourally generated input has dynamical properties strongly differing from those of conventional experimenter-designed stimuli.

## Materials and Methods

### 

#### Stimulus generation and electrophysiology.

The position and orientation of the head of blowflies flying in a cage of 40 × 40 × 40 cm^3^, with images of herbage on its side walls, were recorded using magnetic fields driving search coils attached to the flies [5,8]. Because the fly's compound eye is an integral part of its head, and the visual interior of the cage is known, the visual stimulus encountered by the fly during a flight could be reconstructed. Reconstructions of ten flights of 3.45 s, originating from three different flies, were played back on a panoramic stimulus device [9] at a frame rate of 370 Hz. Proper spatial and temporal prefiltering prevented spatiotemporal aliasing during fast turns [9]. An approximation of the response of the contralateral HSE to the same flight was obtained by presenting a mirrored version of the reconstruction. Intracellular recordings were made from the HSE-cell in the right optic lobe of 1- to 2-d-old female blowflies of *Calliphora vicina,* following standard routines [52] and ensuring careful alignment of the flies' eyes. Results are based on HSE recordings from 14 flies.

#### Data analysis.

Coherence between stimulus and response was calculated as γ^2^_*b*_ = |*P_sr_*|^2^/(*P_ss_P_rr_*) [53], where *P_sr_* is the cross spectral density of stimulus and response, *P_ss_* is the power spectral density of the stimulus, and *P_rr_* is that of the response. The filter *P_sr_*/*P_rr_* reconstructs stimulus from response, and *P_rs_*/*P_ss_*, response from stimulus. Spectra were calculated by periodogram averaging of 50% overlapping data segments, with each periodogram the discrete Fourier transform of a cos^2^-tapered zero-mean data segment of 256 ms, extended by zero-padding to 512 ms. Results were not strongly dependent on segment length. Before segmentation, the response was aligned with the stimulus by shifting it 22.5 ms backwards in time, the approximate latency under the conditions of these experiments. Results were not strongly dependent on shift size. Segments from all flights used as stimulus for a particular cell were included in the periodogram averaging. Bias in the coherence estimate was corrected [54] by γ^2^ = *n*/(*n* − 1)γ^2^_*b*_ − 1/(*n* − 1) , where *n* is the total number of segments. Coherence of the response with two parameters of the stimulus was obtained by first conditioning the second parameter with the first [55], i.e., *s*′_2_ = *s*_2_ − (*P*_21_/*P*_11_)*s*_s_ , where *s*_1_ is the first parameter, and *s*_2_ and *s*′_2_ is the original and conditioned second parameter, respectively; *P*_21_ and *P*_11_ are cross and power spectra of the second and first parameter. Conditioning removes from *s*_2_ the second-order statistical dependence with *s*_1_. With three stimulus parameters (e.g., yaw, sideward, and forward velocity (see Figure 3A), the conditioned third parameter is *s*′_3_ = *s*_3_ − (*P*_32′_/*P*_2′2′_)*s*′_2_ − (*P*_31_/*P*_11_)*s*_1_ , which removes from *s*_3_ the second-order statistical dependence with both *s*_1_ and *s*′_2_ . The order of evaluating parameters does not significantly affect the results for the stimulus parameters used in this study because they are almost uncorrelated.

Masks selecting saccadic segments in stimulus and response were obtained by gating (transmitting) a region surrounding saccades, here defined as peaks (≥500 deg/s) in the total angular velocity of the head. The region was large enough to include all parts of both saccadic stimulus and corresponding response. Saccades that were close together were merged to reduce boundary effects. Edges of the masks were tapered with a 12.5-ms cos^2^ taper to reduce spectral leakage biasing the coherence estimate at high frequencies. The intersaccadic mask, used for suppressing the saccadic stimulus and response, equals one minus the saccadic mask. Masked data consisted of gated data intermitted with blocks of zeroes. Although the mask shapes the power and cross spectra of the masked data, this occurs in a similar way for all spectra in the numerator and denominator of the definition of coherence. Consequently, the mask by itself does not generate coherence for uncorrelated data, which was checked in control computations with uncorrelated noise. The coherence of masked data includes the zero blocks, however, and therefore should be regarded as belonging to the entire masked signal, not just to its intersaccadic part.

The power spectra of Figure 2B were not calculated for the entire masked data because the mask dominates the shape of these spectra, producing a strong peak at the saccade rate, approximately 10 Hz. Instead, we used a routine for calculating the Fourier transform of gapped data [56] and obtained the power spectra by averaging the squared amplitude over segments. The routine ignores all data masked, defined here as points where the intersaccadic mask was smaller than 0.5.

The total optic flow for Figure 1C was calculated by projecting the optic flow onto the local response field of the right HSE cell, i.e., it is weighted according to the cell's local preferred directions and motion sensitivities.

## Supporting Information

Video S1 Flight of 3.45 s, Shown Ten Times Slower than Real Time (at 25 fps Playback Speed) The left panel shows a reconstruction of the flight in a schematic cage, with, for the sake of clarity, the fly rendered three times larger, relative to the cage, than it is in reality. The middle panel shows an enlarged view of the orientational movements of the fly's thorax (blue) and head (red) during the same flight [5,8]. The right panel shows the visual scene, viewed from the centre of the head, during the same flight. It shows a 180 deg fisheye projection, with the centre straight ahead, the far right of the image pointing at 90 deg to the right, and the upper part of the image pointing straight up. The section of 1.5 s length used for Video S2 starts at 1.11 s from the beginning, i.e., approximately at one third of the video. Note the visual consequences of the saccadic yaw changes of thorax and head, and the roll compensation of the head.
(9.9 MB ZIP).

Video S2 Illustration of the OT and OR Control Stimuli The middle panel shows 1.5 s of the video corresponding to the original trajectory, shown 40 times slower than real time, for a patch of 30 deg × 30 deg in a direction in the horizontal plane at 45 deg to the right of the frontal axis of the eye (azimuth 45 deg, elevation 0 deg). This direction is in the middle of the receptive field of the right HSE. The letters *S* and *I* in the title bar signal the time course of the saccadic and intersaccadic masks used for analysing the saccadic and intersaccadic responses, respectively. The left panel shows the OT control in which between saccades (I on), all rotation is removed and only translational optic flow is presented to the fly. The right panel shows the OR control in which between saccades, all translation is removed and only rotational optic flow is presented to the fly. Note that the intersaccadic speeds for sideward translation (original and OT) and rotation (original and OR) are of the same order of magnitude (see the overlapping velocity distributions of yaw and sideward velocity in Figure 2A), whereas the rotations extend to higher temporal frequencies than the translations (see the power spectra of yaw and sideward velocity in Figure 2B). This is reflected in the coding of rotation and translation in the HSE neuron (see the coherences for yaw and sideward velocity in Figure 3A, 3C, and 4B).
(8.8 MB ZIP).

## Abbreviations

HSE: horizontal system equatorial cell
OR: only rotation
OT: only translation
SEM: standard error of the mean
